# High-performance ternary blend polymer solar cells involving both energy transfer and hole relay processes

**DOI:** 10.1038/ncomms8327

**Published:** 2015-06-04

**Authors:** Luyao Lu, Wei Chen, Tao Xu, Luping Yu

**Affiliations:** 1 Department of Chemistry and The James Franck Institute, The University of Chicago, 929 E 57th Street, Chicago, Illinois 60637, USA; 2 Division of Materials Science, Argonne National Laboratory, Argonne, Illinois 60439, USA; 3 Institute for Molecular Engineering, The University of Chicago, 5747 South Ellis Avenue, Chicago, Illinois 60637, USA

## Abstract

The integration of multiple materials with complementary absorptions into a single junction device is regarded as an efficient way to enhance the power conversion efficiency (PCE) of organic solar cells (OSCs). However, because of increased complexity with one more component, only limited high-performance ternary systems have been demonstrated previously. Here we report an efficient ternary blend OSC with a PCE of 9.2%. We show that the third component can reduce surface trap densities in the ternary blend. Detailed studies unravel that the improved performance results from synergistic effects of enlarged open circuit voltage, suppressed trap-assisted recombination, enhanced light absorption, increased hole extraction, efficient energy transfer and better morphology. The working mechanism and high device performance demonstrate new insights and design guidelines for high-performance ternary blend solar cells and suggest that ternary structure is a promising platform to boost the efficiency of OSCs.

## Abstract (web summary)

Ternary blend solar cells offer the potential for high-power conversion efficiencies (PCEs); however their performances can be limited by design complexity. Here, the authors integrate multiple materials into a single junction device with 9.2% PCE and elucidate the mechanisms of enhancement at play.

## Graphical abstract

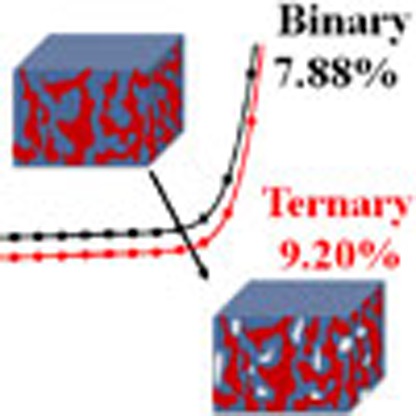

Impressive advances in bulk heterojunction (BHJ) polymer solar cells (PSCs) have been made via material synthesis, device designs, physical measurements and theoretical understanding, which lead to much improved power conversation efficiency (PCE; exceeding 10% in both single junction and tandem cells)^1^,^2^,^3^,^4^. However, despite the improvements achieved, PCEs of the state-of-the-art PSCs are still limited by factors such as insufficient light harvesting and low charge carrier mobilities of the active layer materials, restraining maximum achievable short circuit current density (*J*_sc_) in the devices. A major research effort in the field has been focused on developing new polymers with tunable optoelectronic properties, optimizing device morphology as well as interfacial engineering to enhance exciton dissociation and reduce charge recombination^1^,^5^,^6^,^7^,^8^,^9^,^10^,^11^,^12^,^13^,^14^,^15^,^16^.

Recently, the use of ternary structure-active layer is emerging as a promising strategy to enhance the performances in binary polymer:fullerene BHJ devices^17^,^18^,^19^,^20^. Compared with the binary counterpart, ternary blend PSCs contain one more electron donor or acceptor. In many successful cases, the option of materials as the third component is rather wide open. It could be a quantum dot^21^, a small molecule^22^,^23^ or a polymer^18^,^24^,^25^, which offers considerable opportunities for solar cell optimization. In addition to a complementary absorption with the primary donor material in the solar spectrum and cascade energy levels between highest occupied molecular orbital (HOMO) and lowest unoccupied molecular orbital (LUMO) energy levels of the dominating donor polymer and fullerene acceptor to facilitate charge transport^17^,^26^, our recent results revealed that the ternary blend exhibits improved nanomorphology over the binary device by carefully selecting the third component used. The synergistic effects led to an inspiring PCE of 8.22% for the ternary blend PSCs^27^.

In this work, we use poly-3-oxothieno[3,4-d]isothiazole-1,1-dioxide/benzodithiophene (PID2)^28^ as the additional donor material into binary PSCs based on poly[4,8-bis(5-(2-ethylhexyl)thiophen-2-yl)benzo[1,2-b:4,5-b']dithiophene-co-3-fluorothieno[3,4-b]thiophene-2-carboxylate] (PTB7-Th)^29^ and [6,6]-phenyl C_71_ butyric acid methyl ester (PC_71_BM). We achieve a PCE as high as 9.20% for the ternary device with 20% PID2 content without further device engineering. To our best knowledge, this is the best PCE reported for ternary PSCs to date. Detailed studies show that the enhancement results from a synergistic effect of enhanced light absorption, improved hole mobility, energy transfer between PID2 and PTB7-Th, reduced trap-assisted recombination and improved morphology. The results indicate that the mechanism of the enhancement is completely different from our recent work based on donor polymer PTB7 (ref. 27).

## Results

### Polymer design and characterization

**Figure 1:**
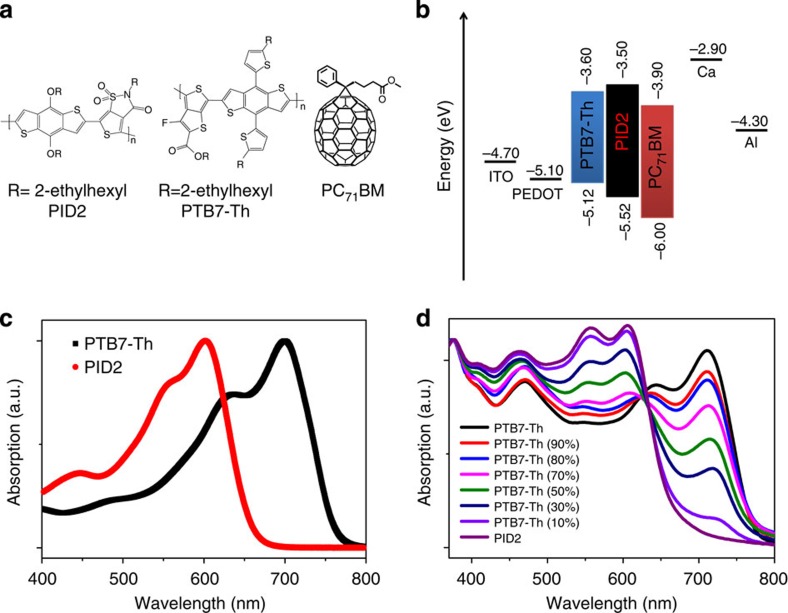
Structures, electrical and optical properties of ternary blends. (**a**) Molecular structures of PID2, PTB7-Th and PC_71_BM. (**b**) Energy levels of materials used in the solar cell device. (**c**) UV–vis absorption spectra of PTB7-Th and PID2 in chloroform. (**d**) UV–vis absorption spectra of PTB7-Th:PID2:PC_71_BM films with different PTB7-Th:PID2 weight ratios.

Chemical structures of PID2, PTB7-Th and PC_71_BM used in the ternary structure are shown in Fig. 1a. HOMO and LUMO energy levels measured by cyclic voltammetry are −5.12, −5.52 and −6.00 and −3.60, −3.50 and −3.90 eV for PTB7-Th, PID2 and PC_71_BM, respectively, which are summarized in Fig. 1b. UV–vis absorption spectra of PTB7-Th and PID2 are presented in Fig. 1c. The maximum absorption for PTB7-Th was at 700 nm and for PID2, the absorption peak appeared at 610 nm. The maximum absorption coefficients for PTB7-Th and PID2 are 28,339 and 31,951 cm^−1^ (Supplementary Fig. 1), respectively. The complementary absorption for the two polymers should be beneficial for charge generation inside the ternary devices. UV–vis absorption spectra of all ternary blend films with different PTB7-Th:PID2 weight ratios were measured and summarized in Fig. 1d. It is clear that incorporation of PID2 gradually increased film absorption strength from 400 to 625 nm while the film absorption capability from 625 to 800 nm was reduced. This is in consistent with the absorption spectra of PID2 and PTB7-Th in Fig. 1c and the absorption coefficients in Supplementary Fig. 1.

### Solar cell characterization

**Figure 2:**
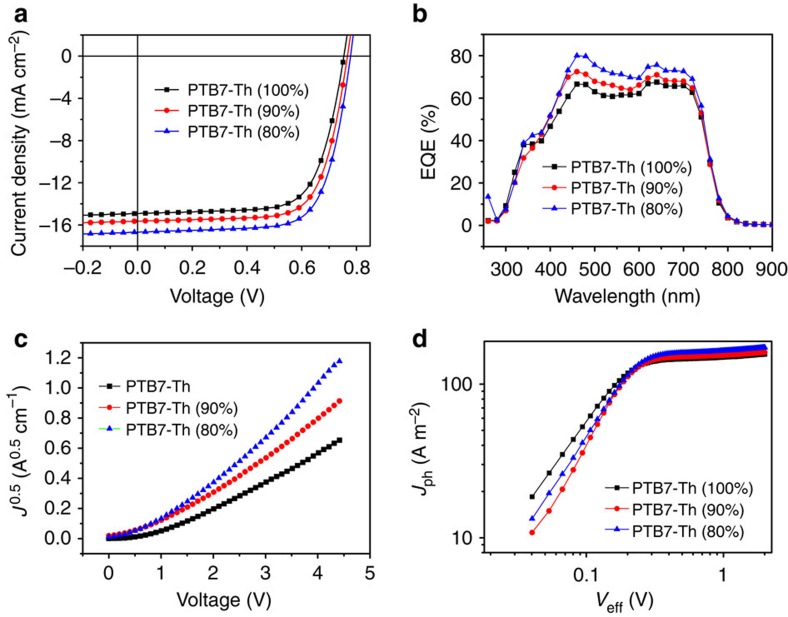
Photovoltaic performance of the ternary devices. (**a**) Current density versus voltage characteristics of PTB7-Th:PID2:PC_71_BM with 0, 10 and 20% PID2 content. (**b**) EQE curves for the ternary devices. (**c**) Hole mobility for the ternary system. (**d**) Photocurrent density versus effective voltage curves.

The photovoltaic performance of the above ternary system was investigated on the basis of the following simple device structure: indium tin oxide (ITO)/poly(3,4-ethylenedioxythiophene):poly(styrenesulphonate) (PEDOT:PSS)/PTB7-Th:PID2:PC_71_BM/Ca/Al. The overall polymer:fullerene ratio was kept at 1:1.5 in this study. The corresponding photovoltaic performance parameters are summarized in Table 1. Figure 2a illustrates the representative current density versus voltage (*J–V*) characteristics of devices with different PTB7-Th:PID2 weight ratios (0, 10 and 20% PID2 incorporation) under simulated AM 1.5 G illumination at 100 mW cm^−2^. Devices with different PID2 concentrations showed consistent thickness around 100 nm. PTB7-Th:PC_71_BM control device started with a *J*_sc_ at 14.92 mA cm^−2^, an open circuit voltage (*V*_oc_) at 0.75 V, a fill factor (FF) at 70.3% and a PCE of 7.88%. By adding 10% of PID2 into the host binary blend, PCE was enhanced to 8.51% with a *J*_sc_ at 15.60 mA cm^−2^, a *V*_oc_ at 0.77 V and a FF at 70.9%. Devices with 20% PID2 incorporation exhibited the best solar cell performance with a *J*_sc_ at 16.68 mA cm^−2^, a *V*_oc_ at 0.78 V and a FF at 70.8%, resulting in a promising PCE of 9.20%. This is more than 15% enhancement in PCE compared with the reference device. We would like to note that the 9.20% PCE is the highest value reported for ternary blend PSCs so far. An average PCE of 8.97±0.16% was achieved over 10 identical devices at this condition with a mean *J*_sc_ at 16.22±0.35 mA cm^−2^, a *V*_oc_ at 0.78±0.01 V and a FF at 70.8±0.14%. PCE of the ternary device was still comparable to the reference cell even if PID2 content was enhanced to 50%. FF remained larger than 70.0% even when PID2 content was increased to 70% and *V*_oc_ increased monotonically with increased PID2 content at all ratios because of deeper HOMO energy levels of PID2 than PTB7-Th. This phenomenon was also observed for many other ternary systems reported^20^,^30^,^31^,^32^ and several possible mechanisms are proposed to explain the increased *V*_oc_, such as the electronic alloy charge-transfer state reported in ref. 33 and parallel-like BHJ concept demonstrated in ref. 18. The existence of co-crystals discussed later in the morphology part rules out the possibility of parallel tandem cell. It is likely that the increased *V*_oc_ with higher PID2 contents is from the electronic alloy charge-transfer state^33^ or the simple mixture of the two distinct charge-transfer states in PTB7-Th and PID2 (ref. 34). Further studies are needed to fully unravel the origin of *V*_oc_ changes in the future. Increasing the loading of PID2 to 90% led to dramatically decreased *J*_sc_ and FF because of the poor performance of PID2 itself. We used devices with 10 and 20% PID2 contents in the following discussions to clarify the effect of PID2 on the ternary system because of their much improved photovoltaic performance than the control device.

### Changes in charge generation and transport properties

External quantum efficiency (EQE) spectra of the ternary devices are shown in Fig. 2b. EQE increased for devices with 10 and 20% PID2 incorporation in a wide wavelength range from 350 to 750 nm. The integrated *J*_sc_ values from EQE spectra for PTB7-Th:PC_71_BM (1.0:1.5), PTB7-Th:PID2:PC_71_BM (0.9:0.1:1.5) and PTB7-Th:PID2:PC_71_BM (0.8:0.2:1.5) devices are 14.52, 15.37 and 16.55 mA cm^−2^, respectively. This is similar to the *J*_sc_ values obtained from *J–V* measurement (the error is within 3%), indicating a good consistency of our solar cell measurements. By comparing with UV–vis absorption spectra in Fig. 1d, it is obvious that enhancement in EQE was not entirely because of absorption changes since addition of PID2 into the host blend actually decreased absorption intensity of PTB7-Th from 625 to 800 nm. As shown in Fig. 1b, HOMO energy levels of PTB7-Th, PID2 and PC_71_BM constructed a cascade structure. As a result, PID2 could act as a hole relay to facilitate hole extraction from PC_71_BM to PTB7-Th after photoexcitation and charge separation^19^,^22^,^27^. This is evidenced by the fact that EQE values in the 440 to 500nm region were enhanced most significantly, where PC_71_BM absorbs.

Hole mobilities of the three devices were then evaluated by space charge-limited current method with the architecture: ITO/PEDOT:PSS/PTB7-Th:PID2:PC_71_BM/MoO_3_/Au. Hole mobility increased from 3.58 × 10^−4 ^cm^2 ^V^−1 ^s^−1^ (PTB7-Th:PC_71_BM, 1:1.5) to 6.18 × 10^−4 ^cm^2 ^V^−1 ^s^−1^ (PTB7-Th:PID2:PC_71_BM, 0.9:0.1:1.5) and finally to 8.82 × 10^−4 ^cm^2 ^V^−1 ^s^−1^ (PTB7-Th:PID2:PC_71_BM, 0.8:0.2:1.5; Fig. 2c). The averaged mobility values over 10 identical devices for the three conditions are (3.33±0.22) × 10^−4 ^cm^2 ^V^−1 ^s^−1^ (0% PID2 loading), (5.96±0.32) × 10^−4 ^cm^2 ^V^−1 ^s^−1^ (10% PID2 loading) and (8.46±0.30) × 10^−4 ^cm^2 ^V^−1 ^s^−1^ (20% PID2 loading), respectively. The improved mobilities helped to partially explain the better *J*_sc_ in the ternary system.

More insight into the effect of PID2 on charge generation and dissociation process was gained by determining the saturation current density (*J*_sat_) and exciton dissociation probabilities (*P(E,T)*) of the ternary solar cells without and with 10 and 20% PID2 incorporation. Figure 2d depicts photocurrent density (*J*_ph_) versus effective voltage (*V*_eff_) characteristics for the three devices. *J*_ph_ is determined as *J*_ph_=*J*_L_−*J*_D_, where *J*_L_ and *J*_D_ are the photocurrent densities under one sun illumination and in the dark, respectively. *V*_eff_ is determined as *V*_eff_=*V*_0_−*V*_a_, where *V*_0_ is the voltage at which *J*_ph_ is 0 and *V*_a_ is the applied bias voltage^35^,^36^. Generally, it is assumed that all the photogenerated excitons are dissociated into free charge carriers at high *V*_eff_ (2 V in this case) and *J*_sat_ is then only limited by maximum exciton generation rate (*G*_max_). As a result, *J*_sat_=*qLG*_max_, where *q* is elementary charge and *L* is the thickness of the active layer^36^. *G*_max_ values for the three devices were 9.79 × 10^27^ m^−3 ^s^−1^ (0% PID2 loading, *J*_sat_=156.8 A m^−2^), 1.01 × 10^28^ m^−3 ^s^−1^ (10% PID2 loading, *J*_sat_=161.5 A m^−2^) and 1.08 × 10^28^ m^−3 ^s^−1^ (20% PID2 loading, *J*_sat_=173.2 A m^−2^), respectively. The average *G*_max_ values over 10 identical devices for the three conditions are (9.69±0.01) × 10^27^ m^−3 ^s^−1^ (0% PID2 loading), (9.93±0.01) × 10^27^ m^−3 ^s^−1^ (10% PID2 loading) and (1.06±0.01) × 10^28^ m^−3 ^s^−1^ (20% PID2 loading), respectively. The increased *G*_max_ with PID2 addition suggested increased overall exciton generations in the ternary devices. This is because of the complementary absorption with a small amount of PID2 incorporation and higher absorption coefficient of PID2 compared with PTB7-Th in the wavelength region below 625 nm. *P(E,T)* is determined from the ratio of *J*_ph_/*J*_sat_ (ref. 37). *P(E,T)* values at short circuit condition for the three devices were 94.8% (0% PID2 loading), 95.3% (10% PID2 loading) and 94.6% (20% PID2 loading), respectively. The incorporation of 10% and 20% PID2 into PTB7-Th:PC_71_BM did not change the overall charge dissociation process. This should be ascribed to the higher LUMO energy level of PID2 than PTB7-Th, which impeded the dissociation of excitons generated by PTB7-Th at PTB7-Th:PID2 interface.

**Figure 3:**
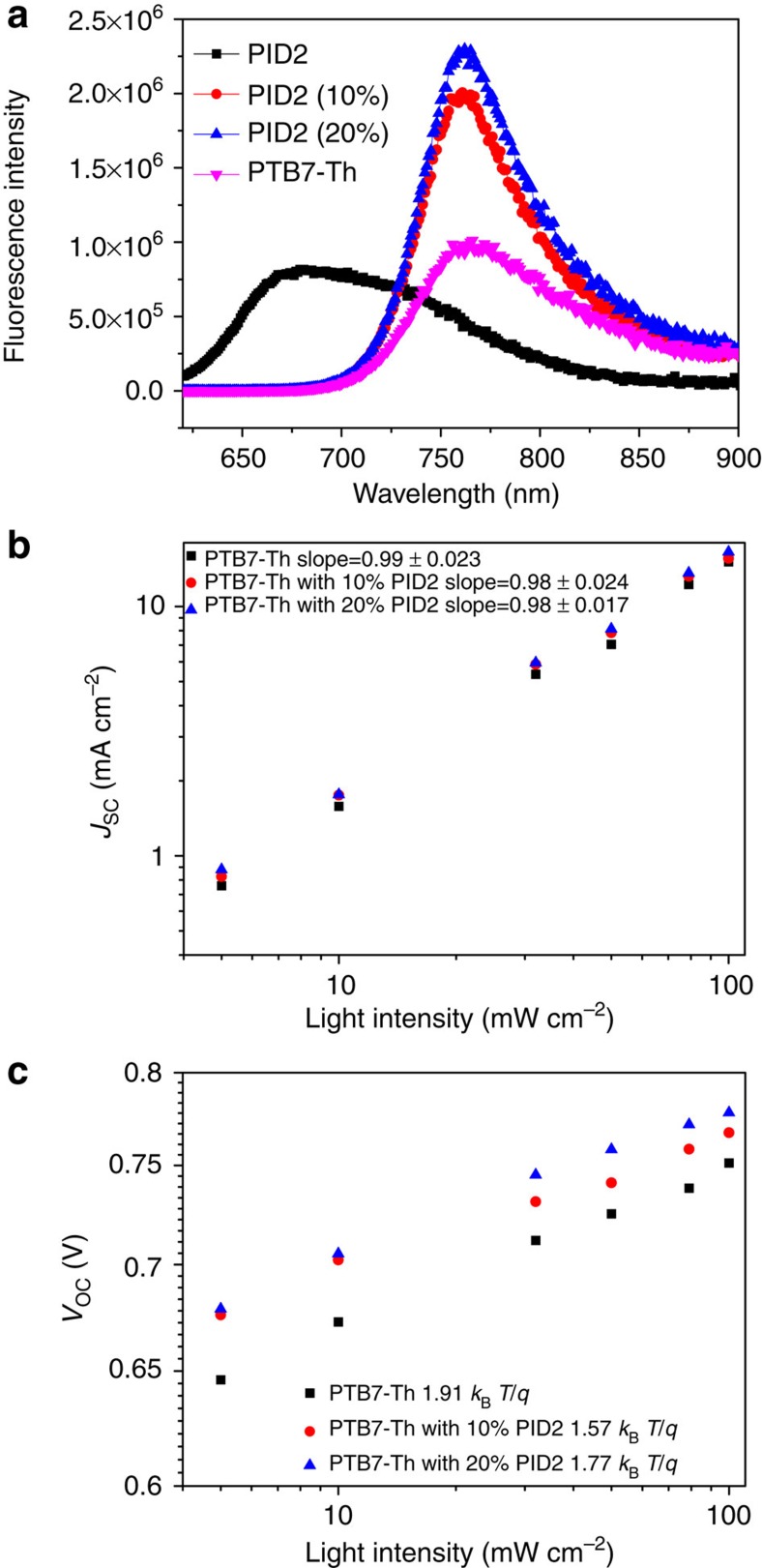
Photoluminescence and recombination study of the ternary devices. (**a**) Photoluminescence spectra of PID2:PTB7-Th:PID2 (0.9:0.1, 0.8:0.2) and PTB7-Th films excited at 610 nm. (**b**) Dependence of *J*_sc_ on light intensity for the ternary system. (**c**) Dependence of *V*_oc_ on light intensity for the ternary system.

The increased exciton generation from PID2 can enhance *J*_sc_ in two possible pathways. Excitons generated by PID2 could directly dissociate at the PID2:PC_71_BM interface and be collected by the electrodes. This is confirmed by the significantly enhanced EQE for ternary devices with (10 and 20%) PID2 content from 500 to 625 nm where PID2 showed improved absorption. Alternatively, energy transfer from photoexcited PID2 to PTB7-Th occurs, followed by charge separation at the PTB7-Th:PC_71_BM interface. This explains the increased EQE from 625 to 750 nm for the ternary devices. Photoluminescence spectra of PTB7-Th only, PID2 only and PTB7-Th with 10 and 20% PID2 films provide evidence for the energy transfer process (Fig. 3a). The photoluminescence spectra of these films were excited at 610 nm, which was the maximum absorption of PID2. PID2 showed a broad emission peak from 650 to 800 nm, which overlapped well with the absorption spectrum of PTB7-Th (Fig. 1c), making energy transfer between PID2 and PTB7-Th favourable. Figure 3a showed that by increasing the amount of PID2 in PTB7-Th, emission of PTB7-Th increased continuously while emission of PID2 was diminished. Although the PID2:PC_71_BM device only gave a *P(E,T)* value at 65.6%, energy transfer from PID2 to PTB7-Th helped exciton dissociation because of a high *P(E,T)* value at 94.8% for PTB7-Th:PC_71_BM device.

### Charge recombination dynamics

The dependence of *J*_sc_ and *V*_oc_ at various light intensities offers deeper insight into the influence of PID2 on recombination process in our ternary system. In principle, *J*_sc_ shows a power-law dependence on light intensity for organic solar cells, which can be expressed as 
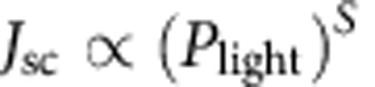
 (refs 38, 39). Here *P*_light_ is light intensity and *S* is the exponential factor. Weak bimolecular recombination in the device would result in a linear dependence of *J*_sc_ on light intensity with *S* value close to 1. Figure 3b illustrates *J*_sc_ as a function of *P*_light_ for PTB7-Th:PC_71_BM (1:1.5), PTB7-Th:PID2:PC_71_BM (0.9:0.1:1.5) and PTB7-Th:PID2:PC_71_BM (0.8:0.2:1.5) devices. The extracted *S* values are 0.99, 0.98 and 0.98 for the three devices, respectively. The similar *S* values indicated that low loading of PID2 has negligible impact on bimolecular recombination process for the ternary system. This should be attributed to the fact that the control device already exhibited very weak bimolecular recombination with an *S* value at 0.99.

Figure 3c shows the relationship between *V*_oc_ and *P*_light_ in our ternary devices. The slope of *V*_oc_ versus log(*P*_light_) helps us to determine the degree of trap-assisted recombination in the devices. A slope at *k*_B_*T*/*q* implies that bimolecular recombination is the dominating mechanism, where *k*_B_ is Boltzmann's constant, *T* is temperature and *q* is elementary charge. As for trap-assisted or Shockley–Read–Hall recombination, a stronger dependence of *V*_oc_ on light intensity with a slope of 2 *k*_B_*T*/*q* is observed^38^,^39^,^40^. In our cases, the PTB7-Th:PC_71_BM device showed a slope of 1.91 *k*_B_*T*/*q* while for PTB7-Th:PID2:PC_71_BM (0.9:0.1:1.5) and PTB7-Th:PID2:PC_71_BM (0.8:0.2:1.5) devices, smaller slopes of 1.57 and 1.77 *k*_B_*T*/*q* were attained. The results indicated that addition of PID2 into the PTB7-Th:PC_71_BM blend reduced interfacial surface trap densities between the active layer materials in the devices, suppressed trap-assisted recombination and contributed to enhanced *J*_sc_.

### Morphology characterization

Influence of PID2 on the morphology of the ternary system was determined by grazing incidence wide-angle X-ray scattering (GIWAXS) and resonant soft X-ray scattering (RSoXS). In the two-dimensional (2D) GIWAXS pattern for each individual polymer film (Supplementary Fig. 2), a broad arc-like scattering arises from the Bragg diffraction of periodic PTB7-Th layers *q*_y_=0.301±0.009** **Å^−1^ and a distinct out-of-plane *π*–*π* stacking peak appears at *q*_z_=1.60±0.06** **Å^−1^, suggesting the preferential face-on conformation, whereas the PID2 bilayer is observed to order in a preferential edge-on orientation with the Bragg diffraction of periodic PID2 layers at *q*_y_=0.346±0.009 Å^−1^ and two off-axis scattering spots located at (± 0.27, 0.38) Å^−1^, similar to the observation in PCDTBT^41^. The 2D GIWAXS patterns also exhibit a scattering peak at *q*_y_ and *q*_z_∼1.3−1.4 Å^−1^ corresponding to the Bragg diffraction of PC_71_BM. Because the full-width at half-maximum (FWHMs) of scattering peak correlates to the nanocrystallite size^42^,^43^, the narrower FWHM of the PID2 layering peak indicates that PID2 in the binary films when blended with PC_71_BM could form larger nanocrystallite sizes than PTB7-Th (Table 2). It is intriguing that, when PTB7-Th and PID2 are blended together, they are not separated with each other, but form co-crystals, as evidenced by the changes in GIWAXS peak positions and their corresponding FWHMs of periodic PTB7-Th and PID2 layers (Supplementary Fig. 3 and Table 2). By incorporating a small amount of PID2 polymers into PTB7-Th:PC_71_BM blend films (PTB7-Th:PID2:PC_71_BM, 0.9:0.1:1.5 and PTB7-Th:PID2:PC_71_BM, 0.8:0.2:1.5), the PID2 polymers had little influence on the co-crystal structures of polymers, which remain similar to PTB7-Th polymers except a slight decrease in the nanocrystallite sizes. Meanwhile, the incorporation of PID2 polymers into the ternary films increases the PC_71_BM nanocrystallite sizes. On further introducing PID2 polymers (PTB7-Th:PID2:PC_71_BM, 0.3:0.7:1.5), a new scattering peak occurs at *q*_y_=0.316±0.009 Å^−1^ in between the scattering of PTB7-Th and PID2 layers, rather than two individual peaks, indicating the intermixing of PTB7-Th and PID2 at the molecular level. The formation of co-crystals would facilitate energy transfer between PTB7-Th and PID2 since the rate constant of fluorescence resonance energy transfer is proportional to (1/*r*)^6^, where *r* is the distance between PTB7-Th and PID2. In addition, the growth of larger PC_71_BM nanocrystallites could benefit charge transport within PC_71_BM domains.

**Figure 4:**
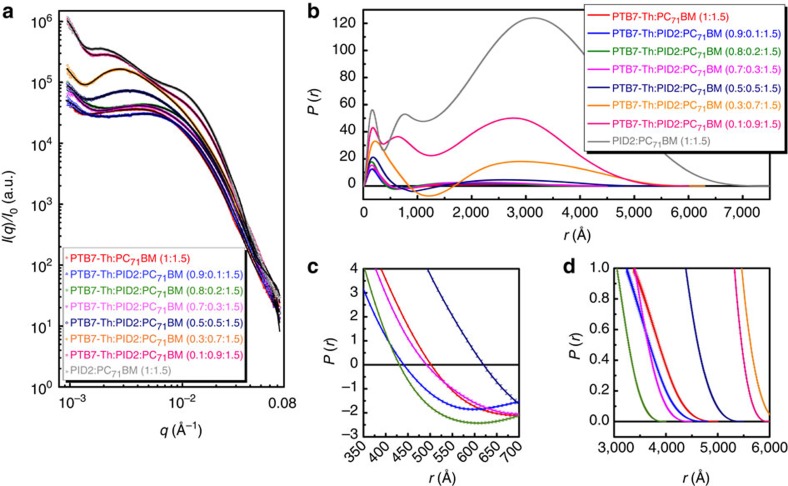
RSoXS characterization of the ternary films. (**a**) RSoXS profiles (open symbols), calculated *I*(*q*) (solid lines), and (**b**) corresponding *P*(*r*) of PTB7-Th:PID2:PC_71_BM ternary systems. (**c**,**d**) The enlarged regimes of *P*(*r*) at the range of *r*=350–700 Å and 3,000–6,000 Å, respectively.

Besides the molecular ordering, another important morphological factor that will determine the photovoltaic performance is how these localized molecular crystals and aggregates form phase-separated domains in the BHJ blend. Figure 4a,b shows the RSoXS profiles (open symbols), the calculated scattering intensities, *I*(*q*), (solid lines) and their corresponding pair distance distribution functions (PDDFs), *P*(*r*), of the ternary PTB7-Th:PID2:PC_71_BM BHJ films, respectively. These ternary PTB7-Th:PID2:PC_71_BM BHJ films show hierarchical nanomorphologies at multiple length scales, in consistence with the previous observations in PTB7:PC_71_BM (ref. 44). As indicated by the zero crossings of *P*(*r*) (Fig. 4c,d), the PID2:PC_71_BM (1:1.5) film exhibits much larger domain sizes than the PTB7-Th:PC_71_BM (1:1.5) film, which correlates with their unsatisfied device performance. When incorporating a small amount of PID2 polymers into PTB7-Th:PC_71_BM films (PTB7-Th:PID2:PC_71_BM, 0.9:0.1:1.5 and PTB7-Th:PID2:PC_71_BM, 0.8:0.2:1.5), such hierarchical nanostructural characteristics are retained with reduced domain sizes. A kink of *P*(*r*) at the length scales of tens of nanometres indicates that the fine domains inside phase-separated domains with hundreds of nanometres scale in these two ternary systems are even smaller than those of PTB7-Th:PC_71_BM (1:1.5). These smaller domains would increase the area of the interfaces between polymer donors and fullerene acceptors, thus promoting exciton dissociation and leading to improved performance. However, because of the poor performance of PID2, overall exciton dissociation probabilities in the ternary blends at 10 and 20% of PID2 incorporation are similar to that in the PTB7-Th:PC_71_BM binary blend. On further increasing the amount of PID2 polymers, larger domains are formed in the ternary blend films and, thereby, result in decreased device efficiency at high PID2 contents. Considering the complicated underlying working mechanism of the ternary BHJ solar cell, morphological characterization helps us to understand the tendency of performance change as a function of PID2 content in our devices.

## Discussion

A highly efficient ternary structure PSC is developed by adding PID2 as the additional donor polymer into the PTB7-Th:PC_71_BM binary device. A PCE of 9.20% was achieved with 20% of PID2 incorporation, which is the highest PCE reported for ternary PSCs. *V*_oc_ of the ternary blends was enhanced with increased PID2 content because of the deeper HOMO energy levels of PID2 compared with PTB7-Th. A high FF at 70.0% was maintained with PID2 content up to 70%. In addition, the combined effects of a complementary light harvesting, efficient energy transfer from PID2 to PTB7-Th, enhanced hole mobility, reduced trap-assisted recombination, increased interfacial areas and better crystallinity of PCBM dramatically improved *J*_sc_ in the ternary systems. Our results demonstrate that the use of ternary structure helps to re-evaluate the potential of many low-performance donor polymers and is a promising way towards developing high-performance PSCs.

## Methods

### Device fabrication

PTB7-Th and PID2 are synthesized according to previous procedures^28^,^29^. The number-averaged molecular weight of PTB7-Th and PID2 are 15.7 and 12.3 kg mol^−1^ with the dispersity index at 2.9 and 2.6, respectively. PTB7-Th, PID2 and PC_71_BM were dissolved in chlorobenzene and 1, 8-diodooctane (97:3, v/v). The overall donor polymer concentration (with or without PID2) was kept constant at 10 mg ml^−1^. The mixed solution was heated at 90 °C overnight. ITO glasses were purchased from thin film devices and sonicated in water, acetone and isopropyl alcohol for 15 min each, followed by ultraviolet ozone irradiation for 60 min. Later, an ∼40-nm-thick PEDOT:PSS layer was spin-coated at 6,000 r.p.m. on ITO glasses and dried at 80 °C in oven for 30 min. Polymer:PCBM solutions were spin-coated with the as-prepared solutions at 2,000 r.p.m. inside glove box. Ca (20 nm) and Al (80 nm) cathodes were thermal-evaporated.

### Solar cell characterization

A solar simulator with a xenon arc lamp (Oriel model 69920) was used to provide 1-sun, AM 1.5G irradiation for the measurement of current density–voltage characteristics. Light intensity of the measurement was calibrated with an NREL-certified monocrystaline silicon reference cell (Newport, 91150 V). The effective area of the *J–V* measurement was determined by masks with well-defined area of 3.14 mm^2^. UV–vis spectra were taken from a UV-2401PC model UV–vis spectrophotometer. EQE was measured with a 250-W Quartz Tungsten Halogen (QTH) lamp as the light source.

### Morphology characterization

GIWAXS measurements were conducted at the 8ID-E beamline^45^ at the Advanced Photon Source, Argonne National Laboratory using X-rays with a wavelength of *λ*=1.6868 Å and a beam size of ∼200 μm (h) and 20 μm (v). RSoXS measurements were achieved at beamline 11.0.1.2 (ref. 46) at the Advanced Light Source, Lawrence Berkeley National Laboratory. Details of GIWAXS and RSoXS measurements can be found in Supplementary Methods.

## Additional information

**How to cite this article:** Lu, L. *et al*. High-performance ternary blend polymer solar cells involving both energy transfer and hole relay processes. *Nat. Commun.* 6:7327 doi: 10.1038/ncomms8327 (2015).

## Supplementary Material

Supplementary Information Supplementary Figures 1-3 and Supplementary Methods and Supplementary References.

## Figures and Tables

**Table 1:** Photovoltaic parameters for PTB7-Th:PID2:PC_71_BM devices with different PID2 concentrations in PTB7-Th.

**PTB7-Th:PID2:PC**_**71**_**BM**	***J***_**sc**_ **(mA cm**^−2^)	***V***_**oc**_ **(V)**	**FF (%)**	**PCE (%)**
1:0:1.5	14.92	0.75	70.3	7.88
0.9:0.1:1.5	15.60	0.77	70.9	8.51
0.8:0.2:1.5	16.68	0.78	70.8	9.20
0.7:0.3:1.5	14.04	0.79	71.6	7.95
0.5:0.5:1.5	13.07	0.80	71.9	7.55
0.3:0.7:1.5	11.50	0.83	70.0	6.68
0.1:0.9:1.5	5.88	0.86	57.5	2.90
0:1:1.5	5.29	0.86	44.3	2.01

FF, fill factor; PCE, power conversion efficiency; PID2, poly-3-oxothieno[3,4-d]isothiazole-1,1-dioxide/benzodithiophenepoly-3-oxothieno[3,4-d]isothiazole-1,1-dioxide/benzodithiophene.

**Table 2:** Summary of 2D GIWAXS parameters of PTB7-Th:PID2:PC_71_BM ternary systems.

**PTB7-Th:PID2:PC**_**71**_**BM**	**Peak at** ***q***_**y**_ **(Å**^−1^)	**FWHM (Å**^−1^)	**Peak at q**_**y**_ **(Å**^−1^)	**FWHM (Å**^−1^)	**Peak at q**_**z**_ **(Å**^−1^)	**FWHM (Å**^−1^)
1:0:1.5	0.301±0.009	0.069	1.35±0.06	0.24	1.37±0.06	0.29
0.9:0.1:1.5	0.301±0.009	0.074	1.35±0.06	0.25	1.36±0.06	0.32
0.8:0.2:1.5	0.306±0.009	0.073	1.33±0.06	0.20	1.36±0.06	0.26
0.7:0.3:1.5	0.303±0.009	0.053	1.32±0.06	0.20	1.36±0.06	0.29
0.5:0.5:1.5	0.303±0.009	0.049	1.29±0.06	0.21	1.33±0.06	0.30
0.3:0.7:1.5	0.316±0.009	0.037	1.32±0.06	0.21	1.35±0.06	0.31
0.1:0.9:1.5	0.337±0.009	0.032	1.31±0.06	0.23	1.35±0.06	0.31
0:1:1.5	0.346±0.009	0.027	1.33±0.06	0.26	1.37±0.06	0.36

2D, two-dimensional; FWHM, full-width at half-maximum.
