# Supplementary material for: High-performance ternary blend polymer solar cells involving both energy transfer and hole relay processes
Source: Nat Commun. 2015 Jun 4;6:7327. doi: 10.1038/ncomms8327 (PMC4468850; doi:10.1038/ncomms8327)
Supplement: Supplementary Information — Supplementary Figures 1-3 and Supplementary Methods and Supplementary References. [file ncomms8327-s1.pdf]

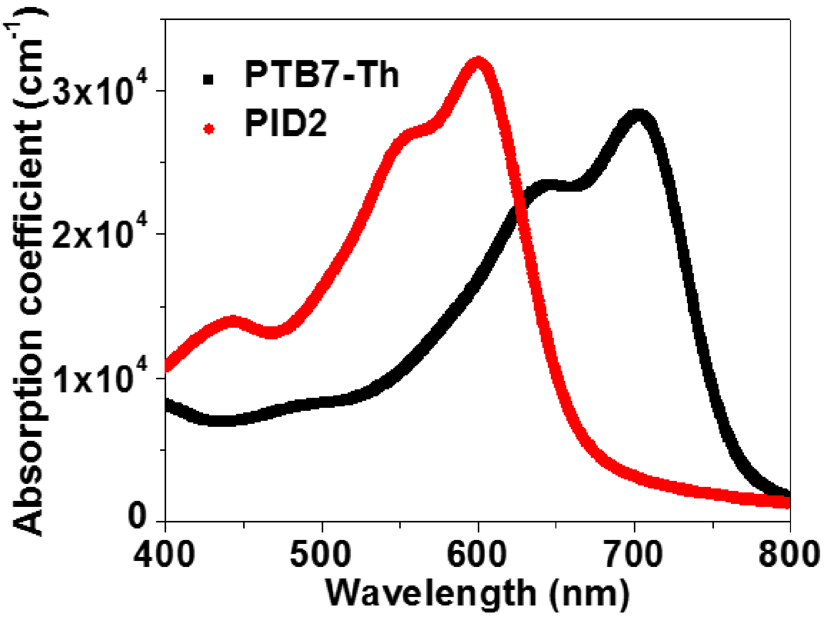

Supplementary Figure 1. Absorption coefficient spectra for PTB7-Th and PID2.

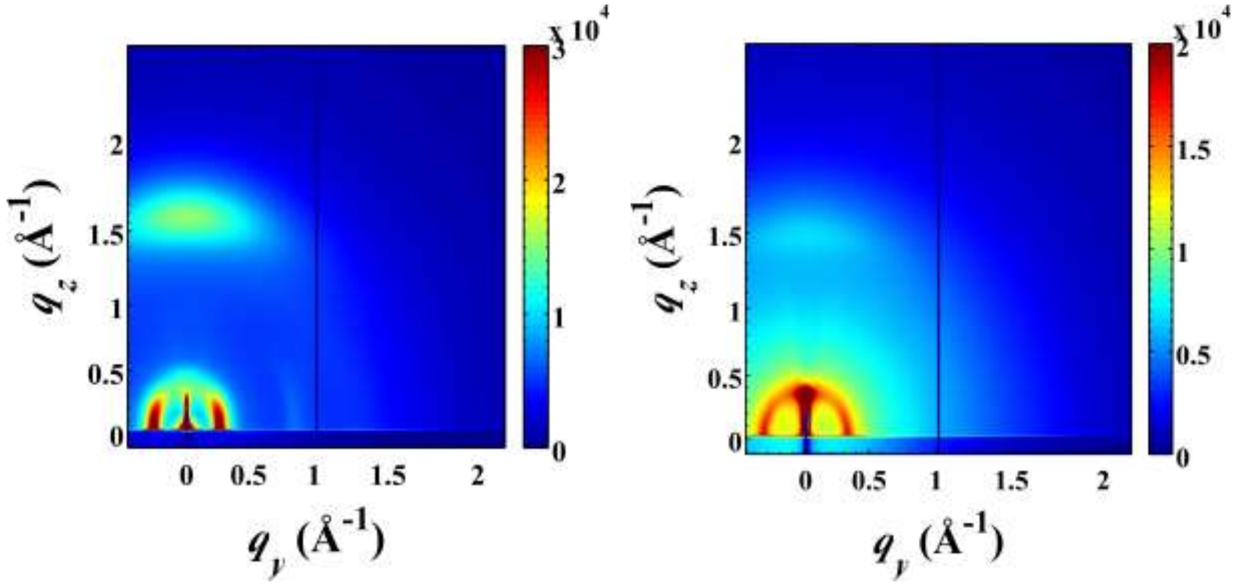

Supplementary Figure 2. 2D GIWAXS patterns of (a) PTB7-Th and (b) PID2 polymers.

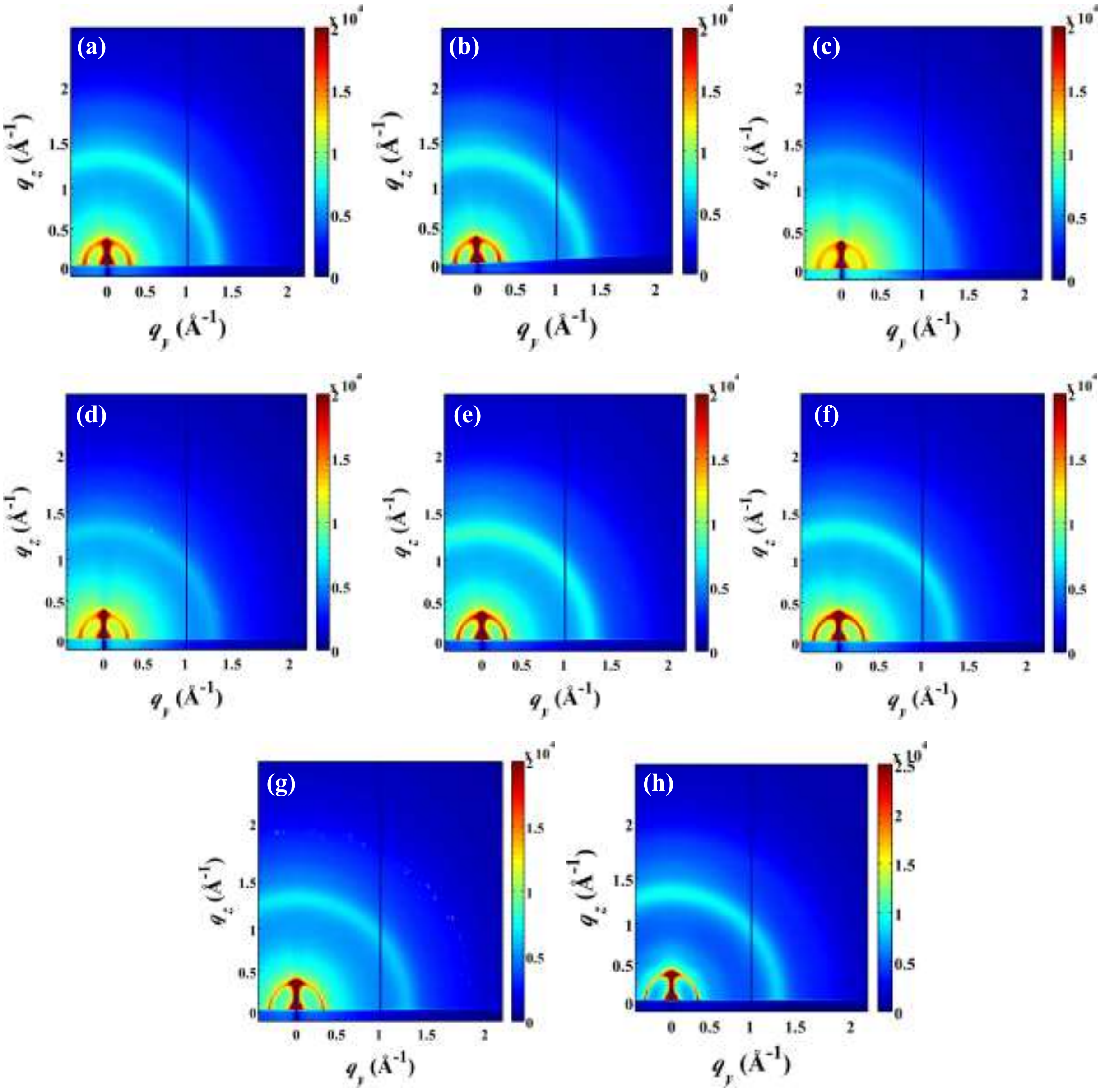

Supplementary Figure 3. 2D GIWAXS patterns of (a) PTB7-Th:$PC_{71}BM$ (1:1.5), (b) PTB7-Th:PID2:$PC_{71}BM$ (0.9:0.1:1.5), (c) PTB7-Th:PID2:$PC_{71}BM$ (0.8:0.2:1.5), (d) PTB7-Th:PID2:$PC_{71}BM$ (0.7:0.3:1.5), (e) PTB7-Th:PID2:$PC_{71}BM$ (0.5:0.5:1.5), (f) PTB7-Th:PID2:$PC_{71}BM$ (0.3:0.7:1.5), (g) PTB7-Th:PID2:$PC_{71}BM$ (0.1:0.9:1.5), and (h) PID2:$PC_{71}BM$ (1:1.5).

Supplementary Methods

Grazing Incidence Wide-Angle X-ray Scattering (GIWAXS)

GIWAXS measurements were performed at the 8ID-E beamline at the Advanced Photon Source (APS), Argonne National Laboratory using x-rays with a wavelength of λ = 1.6868 Å and a beam size of ~200 μm (h) and 20 μm (v).[1] To make the results comparable to those of OPV devices, the samples for the measurements were prepared on PEDOT:PSS modified Si substrates under the same conditions as those used for fabrication of solar cell devices. A 2-D PILATUS 1M-F detector was used to capture the scattering patterns and was situated at 208.7 mm from samples. Typical GIWAXS patterns were taken at an incidence angle of 0.20°, above the critical angles of polymers:$PC_{71}BM$ blends and below the critical angle of the silicon substrate. Consequently, the entire structure of thin films could be detected. The raw scattering intensity was corrected for solid angle correction, efficiency correction for medium (e.g. air) attenuation and detector sensor absorption, polarization correction, flat field correction for removing artifacts caused by variations in the pixel-to-pixel sensitivity of the detector by use of the GIXSGUI package provided by APS, ANL. In addition, the q_y linecut was obtained from a linecut across the reflection beam center, while the q_z linecut was achieved by a linecut at q_y = 0 Å^{-1} using the reflected beam center as zero the silicon substrate. Consequently, the entire structure of thin films could be detected. In addition, the q_y linecut was obtained from a linecut across the reflection beam center. The background of these linecuts was estimated by fitting an exponential function and the parameters of the scattering peaks were obtained through the best fitting using the Pseudo-Voigt type 1 peak function.

Resonant Soft X-ray scattering (RSoXS)

RSoXS transmission measurements were achieved at beamline 11.0.1.2 at the Advanced Light Source (ALS), Lawrence Berkeley National Laboratory.[2] The elliptically polarized undulator (EPU)

source provides high x-ray and full polarization control. The energy of the incident beam can be tuned using a variable-line-space, plane grating monochromator providing soft x-rays in the spectral range from 100 to 1500 eV and the resolving power ($E/\Delta E$) of ~4000. The beam size at the sample position was ~100 μm × 100 μm. The RSoXS chamber was operated at high vacuum (~10^{-7} Torr) and controlled by LabVIEW software developed at ALS. RSoXS was taken with x-ray photon energy of 284.2 eV for the best contrast and sensitivity. A customized designed 4-bounce higher order light suppressor was utilized to suppress higher order light generated from the undulator harmonics and monochromator. The spectral purity of the x-ray photons was higher than 99.99%. Samples for RSoXS measurements were first prepared on a PEDOT:PSS modified Si substrate under the same conditions as those used for fabrication of OPV devices, and then transferred to a 1.5 mm × 1.5 mm, 100 nm thick Si_3N_4 membrane supported by a 5 mm × 5 mm, 200 μm thick Si frame (Norcada Inc.). Single quadrant 2-D scattering patterns were collected on an in-vacuum CCD camera (Princeton Instrument PI-MTE). The scattering patterns were radially averaged and the scattering intensity $I(q)$ in arbitrary units after correcting for background scattering recorded from a blank Si_3N_4 window and normalizing to the incident beam intensity I_0 was plotted against the magnitude of scattering vector, $q=4\pi\sin(\theta/2)/\lambda$ (where θ is the scattering angle and λ is the wavelength of the soft x-rays), on a log-log scale.

Supplementary References

1. Jiang, Z. *et al.* The dedicated high-resolution grazing-incidence X-ray scattering beamline 8-ID-E at the Advanced Photon Source. *J. Synchrotron Rad.* **19**, 627-636 (2012).
2. Gann, E. *et al.* Soft x-ray scattering facility at the Advanced Light Source with real-time data processing and analysis. *Rev. Sci. Instrum.* **83**, 045110-045114 (2012).
